# Autophagy and mTOR Pathways Mediate the Potential Renoprotective Effects of Vitamin D on Diabetic Nephropathy

**DOI:** 10.1155/2020/7941861

**Published:** 2020-05-13

**Authors:** Suzan A. Khodir, Rehab M. Samaka, Omnia Ameen

**Affiliations:** ^1^Physiology Department, Faculty of Medicine, Menoufia University, Shebeen El-Kom, Menoufia, Egypt; ^2^Pathology Department, Faculty of Medicine, Menoufia University, Shebeen El-Kom, Menoufia, Egypt

## Abstract

**Introduction:**

Not only is diabetic nephropathy (DN) the most common cause of end-stage renal disease worldwide, but it also increases the risk of mortality up to fourteen times compared to normoalbuminuric diabetic patients.

**Aim:**

The aim of the current study was the evaluation of the renoprotective effects of vitamin D in DN and the possible interplay between autophagy and mTOR pathways.

**Materials and Methods:**

Fifty male Wistar albino rats were divided (10/group) into control, DN group, insulin-treated DN group, vitamin D-treated DN group, and combined insulin and vitamin D-treated DN group. Assessments of systolic blood pressure, albuminuria, creatinine clearance, serum glucose, insulin, urea, creatinine, inflammatory cytokines, oxidative stress markers, and rat kidney gene expression of mTOR were performed. Histopathological and immunohistochemical assessments of autophagy marker LC3 in rat kidneys were also performed.

**Results:**

DN was associated with significant increases in SBP, urinary albumin, serum glucose, urea, creatinine, inflammatory cytokines, MDA, and mTOR gene expression (*P* < 0.05). However, there was significant decrease in creatinine clearance, serum insulin, GSH, and H score value of LC3 when compared with control group (*P* < 0.05). The combination of insulin and vitamin D treatment significantly restored DN changes when compared with the other treated groups, except in oxidative stress markers where there was an insignificant difference between the combination-treated and insulin-treated groups (*P* > 0.05).

**Conclusion:**

It has been concluded that vitamin D is a potent adjuvant therapy in treatment of DN via downregulation of mTOR gene expression, stimulation of autophagy, and antioxidant, anti-inflammatory, and hypotensive effects.

## 1. Introduction

Diabetic nephropathy (DN) is one of the major microvascular complications of diabetes and a major cause of end-stage kidney disease in the world. About 25–40% of diabetic patients develop DN within 20–25 years of the onset of their diabetes [1]. It causes glomerular damage along with proteinuria and subsequent tubule-interstitial lesions, leading to end-stage renal disease [2]. DN accounts for the high levels of disability and the high mortality rates in diabetic patients [1].

The production of reactive oxygen species (ROS) in the kidney is enhanced by high glucose concentration [3]. Impairment of the oxidant/antioxidant equilibrium results in oxidative stress in numerous pathological conditions, including DN, which leads to cellular damage [1]. Increase of advanced renal glycation end products (AGEs) and excessive secretion of inflammatory cytokines have been shown to be associated with DN [4]. Mammalian target of rapamycin (mTOR) is a protein kinase that is broadly expressed in multiple organs and cells, including podocytes and proximal convolute tubule cells. A number of studies have reported that mTOR participates in the hyperproliferation of mesangial cells associated with DN [5].

Autophagy is a bulk degradation process involved in the clearance of damaged proteins and organelles [6]. Under basal conditions, podocytes have a high constitutive level of autophagy. Podocyte-specific autophagy-deficient mice developed podocyte loss and massive proteinuria [7]. Microtubule-associated protein 1 light chain 3 (LC3) is a soluble protein that is proteolytically modified by a C-terminal cleavage to generate a form (LC3-I) that is subsequently conjugated to phosphatidylethanolamine (PE) to produce LC3–PE (or LC3-II), which is recruited to phagophore membranes. Meanwhile, the high conversion of LC3-I into LC3-II reflects either a high autophagic flux or a blockade in autolysosomal degradation. Accordingly, LC3 is an autophagy regulator gene. It is considered as standard for autophagosome formation [8].

The incidence of diabetic kidney disease continues to increase and many patients with DN experience progressive kidney function decline resulting in end-stage kidney disease [9]. Hence, there is a critical need to further understand the pathogenesis of DN in order to identify new therapeutic targets and improve clinical management.

Numerous epidemiological studies have suggested that vitamin D may have a role in defense against diabetes [10]. In the kidney, vitamin D may be important for maintaining podocyte health, preventing epithelial-to-mesenchymal transformation, and suppressing inflammation and oxidative stress [11]. Also, active vitamin D3 could effectively reduce the renal fibrosis and protect the renal function in DN rat model [12]. Recent experimental data suggest that vitamin D protects podocytes by targeting multiple pathways, including autophagy [13] and mTOR [5].

Despite the importance of vitamin D in glucose homeostasis [14], few studies assess the potential effect of its combination with other antidiabetic drugs on DN. Considering the high prevalence of both vitamin D deficiency and diabetes mellitus and to clarify their relationship, this study was designed to shed light on the potential effects of vitamin D on DN, and the possible underlying mechanisms and interplays between autophagy and mTOR pathways.

## 2. Materials and Methods

### 2.1. Animals

The study was conducted on fifty Wistar albino rats weighing 200–250 gram after obtaining approval from the Research Ethical Committee, Faculty of Medicine, Menoufia University, Egypt. Experimental procedures followed the Guide for the Care and Use of Laboratory Animals, 8th edition (National Research Council 2011). The rats were housed in wire mesh cages (80 × 40 × 30 cm). Prior to experiment, all animals were conditioned for 2 weeks at constant environmental conditions and 12 : 12-h light/dark cycle. They were given free access to chow and water throughout the study period.

Diabetes was induced by a single intraperitoneal (i.p.) injection of streptozotocin (STZ) (60 mg/kg in 0.2 ml of 10 mmol/L citrate buffer (pH 4.5), Sigma-Aldrich Chemical Co., USA) [15]. The development of diabetes was confirmed after 48 h of the STZ injection. The animals with fasting blood glucose level more than 200 mg/dl were considered as diabetic and selected for the experiment. Fifty male Wistar albino rats were randomly divided into the following groups (10 rats per group):control group: rats received single i.p. injection of 10 mmol/L citrate buffer (pH 4.5) and 10 ml of peanut oil administered intragastrically once daily for 8 weeks; diabetic nephropathy group (DN group): diabetes was induced, and then 10 ml of peanut oil was administered intragastrically once daily for 8 weeks; insulin-treated DN group: diabetes induction was followed by administration of insulin (0.75 IU/100 gm subcutaneously once daily for 8 weeks, Sigma-Aldrich Co., USA) [16] and 10 ml of peanut oil intragastrically once daily for 8 weeks; vitamin D-treated DN group: diabetes induction was followed by administration of vitamin D (calcitriol (Rocaltrol), 2.5 *μ*g/kg, dissolved in 10 ml of peanut oil intragastrically once daily for 8 weeks; Rocaltrol was purchased from Shanghai Roche Medical Co., Ltd.) [1]; combined insulin and vitamin D-treated DN group: diabetes induction was followed by administration of a combination of insulin and vitamin D at the same doses. Rats developed DN after 5 weeks from being diabetic [17].

At the end of the study, all rats were weighed, subjected to arterial blood pressure assessment, and then housed individually in metabolic cages for 24-hour urine collection. The urine was collected in graded tubes, and volume was measured and then centrifuged to separate debris. Blood samples, then, were withdrawn for biochemical analysis. Finally, all animals were sacrificed, and right kidneys were dissected and kept frozen at −80°C to estimate the gene expression, while the left kidneys were dissected and fixed in 10% neutralized formalin solution for histopathological and immunohistochemical examination.

### 2.2. Experimental Procedures

#### 2.2.1. Measurement of Systolic Blood Pressure

Systolic blood pressure was determined by means of a rat-tail pressure detecting equipment connected to a pneumatic transducer (Harvard Apparatus Ltd., Aden Berge, England).

#### 2.2.2. Measurements of Albuminuria and Creatinine Clearance

Urinary albumin level was assessed using Microalbuminuria ELISA (Exocell Inc., Philadelphia, USA). The creatinine clearance (ml/min) was calculated by using the following formula: creatinine clearance = *U* × *V*/*P* (*U*: creatinine concentration in urine (mg/dl), *V*: volume of urine per minute (ml/min), *P*: creatinine concentration in plasma (mg/dl)) [18].

#### 2.2.3. Blood Samples Collection

Rats were fasted overnight, and then blood samples were drawn retro-orbitally via heparinized microcapillary tubes. The blood was allowed to coagulate for 30 minutes at room temperature and then centrifuged at 2000 rpm for 10 min to separate serum samples. Samples were stored at −20°C.

#### 2.2.4. Biochemical Analysis

Serum samples were used for estimation of glucose (Diamond Diagnostic, Egypt) and insulin (DRG Instruments GmbH, Germany). Urea, creatinine, malondialdehyde (MDA), and glutathione (GSH) levels were measured using the conventional colorimetric assay (QuantiChrom™, BioAssay Systems, USA). Interleukin-6 was determined using ELISA kit (R&D Systems Inc., USA; MyBioSource Company, San Diego, USA), and tumor necrosis factor alpha (TNF-*α*) was quantified using ELISA kit (Quantikine, Abcam Company, Cambridge, UK). All of the above assays were carried out according to the manufacturers' instructions.

#### 2.2.5. Quantitative Assay of mTOR Gene Expression Using Reverse Transcriptase Polymerase Chain Reaction (RT-PCR) Technique

Kidney samples were prepared for total RNA isolation using Qiagen RNeasy Plus Universal Kit, USA, and then RNA quality and purity were assured. RNA was stored in −80°C till used, and then the first step was cDNA synthesis using QuantiTect Reverse Transcription Kit, Qiagen, USA, using Applied Biosystems 2720 Thermal Cycler (Singapore) for only one cycle. GAPDH primers were used in RT-PCR reaction as RNA loading control. The second step was cDNA amplification: the cDNA was used in SYBR Green Based Quantitative Real-Time PCR for Relative Quantification (RQ) of mTOR gene expression by SensiFAST^™^ SYBR Lo-ROX Kit, USA, using the following designed primers (Midland, Texas): Forward, *5-TTGCCAACTACC TTCGGAACC-3;* Reverse*, 5-TCA CGGAGAACGA GGACAGC-3*. Finally, data analysis was conducted using the Applied Biosystems 7500 software version 2.0.1. The RQ of mTOR gene expression was performed using comparative ∆∆Ct method where the amount of the target (mTOR) mRNA is normalized to an endogenous reference gene (GAPDH) and relative to a control.

#### 2.2.6. Histopathology Assessment

The right kidney for each rat was dissected and preserved in 10% formalin solution. The specimens were sent to Pathology Department, Faculty of Medicine, Menoufia University, for routine processing and preparation of H&E stained slides. H&E stained sections were examined to study the histopathological changes. Microscopic examinations were carried out for all groups. Unintentional bias was prevented by coding rats' tissue samples. Kidney sections were assessed for glomerular parameters: cellularity, patency of capillaries, mesangial proliferation, and mesangial matrix status; tubular parameters: atrophy, tubulitis, hydropic degeneration, crystals, cast formation, and peritubular fibrosis; and interstitial tissue changes: edema, fibrosis, and inflammatory cellular infiltrates.

#### 2.2.7. Renal Immunohistochemical Staining with LC3

Immunohistochemical staining was performed using rabbit polyclonal antibody to light chain 3 (LC3) (Ab: 100 *μ*g/ml) (Cat. No. YPA1340; Chongqing Biospes Co., Ltd. Chongqing, China). It was received in a single vial containing 1 ml of concentrated antibody and diluted 1 : 150. Immunohistochemical staining was performed using the Universal Dako Labelled Streptavidin–Biotin-2 system. The primary antibody was applied on the slides and incubated overnight at room temperature in humid chamber. Finally, the detection of bound antibody was accomplished using a modified labeled avidin-biotin (LAB) reagent for 20 minutes and then phosphate-buffered saline (PBS) wash. A 0.1% solution of diaminobenzidine was used for 5 minutes as a chromogen. Slides were counterstained with Mayer's hematoxylin for 5–10 minutes. Negative control slides were prepared by omitting the primary antibodies from the staining procedure. Positive control was normal colon [19].

Immunohistochemical interpretation of LC3 was assessed in all studied groups: any cytoplasmic staining of LC3 was considered positive. Distribution of positivity was assessed (glomerular or tubular). Percentage of positive cells and intensity of staining (mild, moderate, or strong) were assessed to calculate histoscores (H score) as follows: 3 (strong intensity) × % + 2 (moderate intensity) × % + 1 (mild intensity) × %. The score ranges between 0 and 300 [20]. Then the H score was divided into 2 categories (low or high) using median.

### 2.3. Statistical Analysis

Results are expressed as mean ± standard deviation (SD). Analysis of variance (ANOVA) was used for statistical analysis of the different groups followed by post hoc Tukey test, using Origin® software and the probability of chance (*P* values). *P* values <0.05 were considered significant.

## 3. Results

### 3.1. Fasting Serum Glucose and Insulin

**Figure 1:**
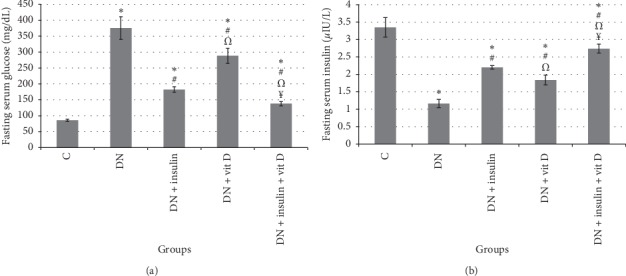
(a) Fasting serum glucose (mg/dL); (b) fasting serum insulin (*μ*IU/L) in all studied groups (∗: significant when compared to C group, #: significant when compared to DN group, Ω: significant when compared to DN + insulin group, ¥: significant when compared to DN + vit D group). Data are shown as means + SD (*n* = 10). ANOVA was used to make group comparisons. Significance: *P* < 0.05.

The mean value of serum glucose level in DN group was significantly higher than that in control group (375 ± 35.5 vs 85.83 ± 3.43 mg/dL, respectively; *P* < 0.05). Serum glucose levels in insulin-treated, vitamin D-treated, and combined insulin and vitamin D-treated groups were significantly lower than that in DN group (182.33 ± 8.55, 288.33 ± 24.0, 138 ± 6.72 mg/dL, respectively; *P* < 0.05) but still significantly higher than that in control group (*P* < 0.05). Serum glucose level of combined insulin and vitamin D-treated group was significantly lower than that in insulin-treated and vitamin D-treated groups (*P* < 0.05). However, serum glucose level of insulin-treated group was significantly lower than that of vitamin D-treated group (*P* < 0.05). The mean value of serum insulin level in DN group was significantly lower than that in control groups (1.16 ± 0.12 vs 3.35 ± 0.28 *μ*IU/L, respectively; *P* < 0.05). Serum insulin levels in insulin-treated, vitamin D-treated, and combined insulin and vitamin D-treated groups were significantly higher than that in DN group (2.20 ± 0.05, 1.84 ± 0.14, 2.74 ± 0.13 *μ*IU/L, respectively; *P* < 0.05) but significantly lower than that in control group (*P* < 0.05). Serum insulin level of combined insulin and vitamin D-treated group was significantly higher than those in insulin-treated and vitamin D-treated groups (*P* < 0.05). Serum insulin level of insulin-treated group was significantly higher than that in vitamin D-treated group (Figures 1(a) and 1(b)).

### 3.2. Renal Function Tests

#### 3.2.1. Urinary Albumin and Serum Urea

**Figure 2:**
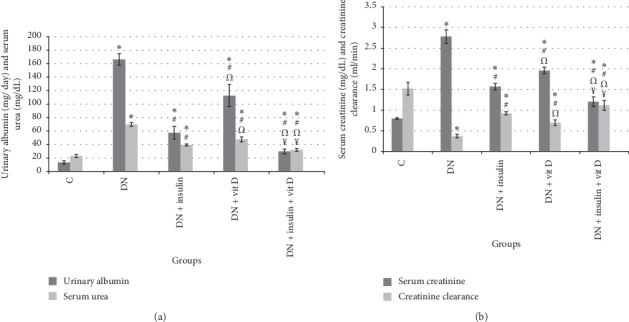
(a) Urinary albumin (mg/day) and serum urea (mg/dL). (b) Serum creatinine (mg/dL) and creatinine clearance (ml/min) in all studied groups (*∗*: significant when compared to C group, #: significant when compared to DN group, Ω: significant when compared to DN + insulin group, ¥: significant when compared to DN + vit D group). Data are shown as means + SD (*n* = 10). ANOVA was used to make group comparisons. Significance: *P* < 0.05.

The mean value of urinary albumin level was significantly higher in DN group than in control group (166.17 ± 8.16 vs 13.50 ± 2.43 mg/day, respectively; *P* < 0.05). Urinary albumin levels in insulin-treated, vitamin D-treated, and combined insulin and vitamin D-treated groups were significantly lower than that in DN group (57.50 ± 9.35, 112.50 ± 16.36, 29.67 ± 3.78 mg/day, respectively; *P* < 0.05) but significantly higher than that in control group (*P* < 0.05). Urinary albumin level of combined insulin and vitamin D-treated group was significantly lower than those in insulin-treated and vitamin D-treated groups. However, urinary albumin level of insulin-treated group was significantly lower than that in vitamin D-treated group. The mean value of serum urea was significantly higher in DN group than in control group (69.67 ± 2.62 vs 23.03 ± 2.12 mg/dL, respectively; *P* < 0.05). Serum urea levels in insulin-treated, vitamin D-treated, and combined insulin and vitamin D-treated groups were significantly lower than that in DN group (39.52 ± 1.40, 47.75 ± 3.65, 32.03 ± 1.83 mg/dL, respectively; *P* < 0.05) and significantly higher than that in control group (*P* < 0.05). Serum urea level of combined insulin and vitamin D-treated group was significantly lower than those in insulin-treated and vitamin D-treated groups (*P* < 0.05). Serum urea level of insulin-treated group was significantly lower than that in vitamin D-treated group (Figure 2(a)).

#### 3.2.2. Serum Creatinine and Creatinine Clearance

The mean value of serum creatinine level in DN group was significantly higher than that in control group (2.78 ± 0.164 vs 0.8 ± 0.019 mg/dl, respectively; *P* < 0.05). Serum creatinine levels in insulin-treated, vitamin D-treated, and combined insulin and vitamin D-treated groups were significantly lower than that in DN group (1.57 ± 0.078, 1.96 ± 0.08, 1.205 ± 0.12 mg/dl, respectively; *P* < 0.05) and still significantly higher than that in control group (*P* < 0.05). Serum creatinine level of combined insulin and vitamin D-treated group was significantly lower than those in insulin-treated and vitamin D-treated groups (*P* < 0.05). Serum creatinine level of insulin-treated group was significantly lower than that in vitamin D-treated group. Creatinine clearance value in DN group was significantly lower than that in control group (0.373 ± 0.044 vs 1.52 ± 0.15 ml/min, respectively; *P* < 0.05). Creatinine clearance values in insulin-treated, vitamin D-treated, and combined insulin and vitamin D-treated groups were significantly higher than that in DN group (0.9250 ± 0.037, 0.698 ± 0.07, 1.115 ± 0.119 ml/min, respectively; *P* < 0.05) and significantly lower than that in control group (*P* < 0.05). Creatinine clearance value of combined insulin and vitamin D-treated group was significantly higher than those in insulin-treated and vitamin D-treated groups (*P* < 0.05). Creatinine clearance value of insulin-treated group was significantly higher than that of vitamin D-treated group (Figure 2(b)).

### 3.3. Serum MDA and GSH

**Figure 3:**
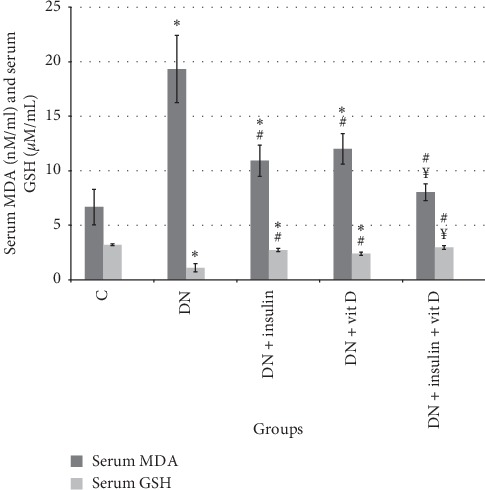
Serum MDA (nM/mL) and serum GSH (*μ*M/mL) in all studied groups (*∗*: significant when compared to C group, #: significant when compared to DN group, Ω: significant when compared to DN + insulin group, ¥: significant when compared to DN + vit D group). Data are shown as means + SD (*n* = 10). ANOVA was used to make group comparisons. Significance: *P* < 0.05.

The mean value of serum MDA level in DN group was significantly higher than that in control group (19.33 ± 3.08 vs 6.67 ± 1.63 nM/mL, respectively; *P* < 0.05). Serum MDA levels in insulin-treated, vitamin D-treated, and combined insulin and vitamin D-treated groups were significantly lower than that in DN group (10.92 ± 1.43, 12 ± 1.41, 8.02 ± 0.77 nM/mL, respectively; *P* < 0.05). Serum MDA level of combined insulin and vitamin D-treated group was significantly lower than that of vitamin D-treated (*P* < 0.05), but there was insignificant difference when compared with insulin-treated and control groups (*P* > 0.05). Also, there was insignificant difference in serum MDA level between insulin-treated group and vitamin D-treated group (*P* > 0.05). Serum MDA levels of vitamin D-treated and insulin-treated groups were significantly higher than that of the control group (*P* < 0.05). Serum GSH in DN group was significantly lower than that in control group (1.1 ± 0.17 vs 3.21 ± 0.08 *μ*M/mL, respectively; *P* < 0.05). Serum GSH levels in insulin-treated, vitamin D-treated, and combined insulin and vitamin D-treated groups were significantly higher than that in the DN group (2.72 ± 0.154, 2.39 ± 0.15, 2.96 ± 0.162 *μ*M/mL, respectively). Serum GSH level of combined insulin and vitamin D-treated group was significantly higher than that of vitamin D-treated (*P* < 0.05), but there was insignificant difference when compared with insulin-treated and control groups (*P* > 0.05). There was insignificant difference in serum GSH level between insulin-treated group and vitamin D-treated group (*P* > 0.05). Serum GSH levels of vitamin D-treated and insulin-treated groups were significantly lower than that of the control group (Figure 3).

### 3.4. Serum IL-6 and TNF-*α*

**Figure 4:**
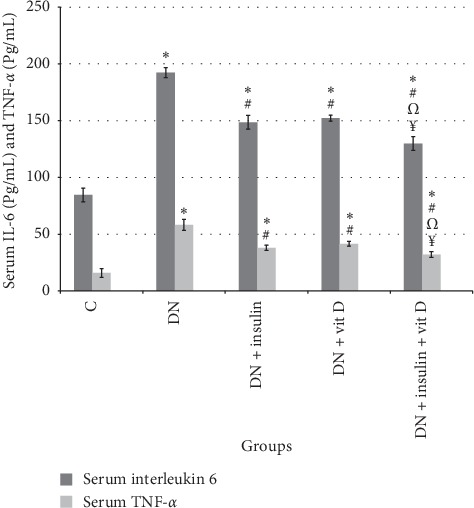
Serum IL-6 (pg/mL) and TNF-*α* (pg/mL) in all studied groups (*∗*: significant when compared to C group, #: significant when compared to DN group, Ω: significant when compared to DN + insulin group, ¥: significant when compared to DN + vit D group). Data are shown as means + SD (*n* = 10). ANOVA was used to make group comparisons. Significance: *P* < 0.05.

Serum IL-6 level in DN group was significantly higher than that in control group (192.37 ± 4.38 vs 84.55 ± 6.07 pg/mL, respectively; *P* < 0.05). Serum IL-6 levels in insulin-treated, vitamin D-treated, and combined insulin and vitamin D-treated groups were significantly lower than that in DN group (148.53 ± 6.82, 152.22 ± 2.74, 129.83 ± 6.04 pg/mL, respectively; *P* > 0.05) and significantly higher than that in control group (*P* < 0.05). Serum IL-6 level in combined insulin and vitamin D-treated group was significantly lower than those in insulin-treated and vitamin D-treated groups. There was insignificant difference in serum IL-6 level between insulin-treated and vitamin D-treated groups (*P* > 0.05). Serum TNF-*α* in DN group was significantly higher than that in control group (58.22 ± 4.78 vs 15.83 ± 3.89 pg/mL, respectively; *P* < 0.05). Serum TNF-*α* levels in insulin-treated, vitamin D-treated, and combined insulin and vitamin D-treated groups were significantly lower than that in DN group (38.06 ± 2.44, 41.58 ± 1.96, 32.1 ± 2.52 pg/mL, respectively; *P* < 0.05) and significantly higher than that in control group (*P* < 0.05). Serum TNF-*α* level of combined insulin and vitamin D-treated group was significantly lower than those of insulin-treated and vitamin D-treated groups. There was insignificant difference in serum TNF-*α* level between insulin-treated and vitamin D-treated groups (*P* > 0.05) (Figure 4).

### 3.5. SBP

**Figure 5:**
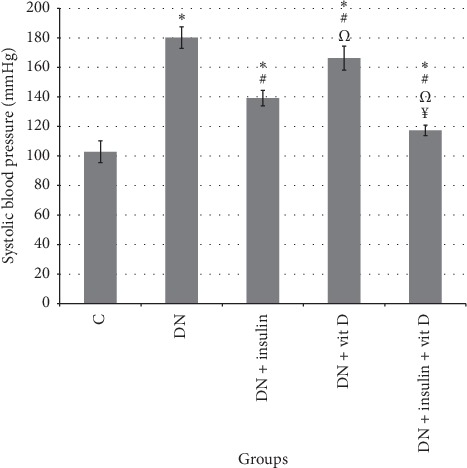
Systolic blood pressure (mmHg) in all studied groups (*∗*: significant when compared to C group, #: significant when compared to DN group, Ω: significant when compared to DN + insulin group, ¥: significant when compared to DN + vit D group). Data are shown as means + SD (*n* = 10). ANOVA was used to make group comparisons. Significance: *P* < 0.05.

The mean value of SBP in DN group was significantly higher than that in control group (180.17 ± 7.31 vs 102.8 ± 7.31 mmHg, respectively; *P* < 0.05). SBP values in insulin-treated, vitamin D-treated, and combined insulin and vitamin D-treated groups were significantly lower than that in DN group (139.17 ± 5.27, 166.33 ± 8.17, 117.33 ± 3.56 mmHg, respectively; *P* < 0.05) and significantly higher than that in control group (*P* < 0.05). SBP value in combined insulin and vitamin D-treated group was significantly lower than those in insulin-treated and vitamin D-treated groups. SBP in insulin-treated group was significantly lower than that in vitamin D-treated group (Figure 5).

### 3.6. mTOR Gene Expression

**Figure 6:**
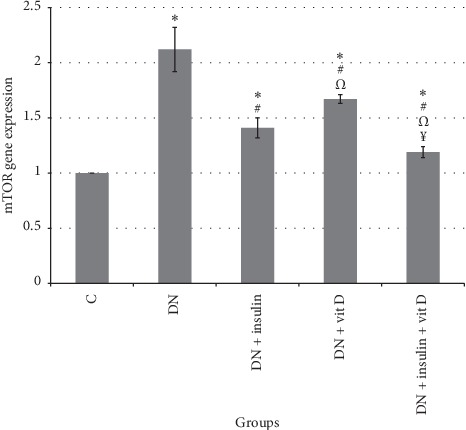
mTOR gene expression in all studied groups (*∗*: significant when compared to C group, #: significant when compared to DN group, Ω: significant when compared to DN + insulin group, ¥: significant when compared to DN + vit D group). Data are shown as means + SD (*n* = 10). ANOVA was used to make group comparisons. Significance: *P* < 0.05.

Expression of mTOR gene in DN group was significantly upregulated compared to control group (2.12 ± 0.21 vs 1 ± 0, respectively; *P* < 0.05). However, expression of mTOR genes in insulin-treated, vitamin D-treated, and combined insulin and vitamin D-treated groups was significantly downregulated compared to DN group (1.41 ± 0.09, 1.67 ± 0.05, 1.19 ± 0.05, respectively; *P* < 0.05) but was significantly higher than that in control group. Expression of mTOR gene in combined insulin and vitamin D-treated group was significantly downregulated compared to insulin-treated and vitamin D-treated groups. Expression of mTOR gene in insulin-treated group was significantly downregulated compared to vitamin D-treated group (Figure 6).

### 3.7. Histopathological Assessment of Different Groups

**Figure 7:**
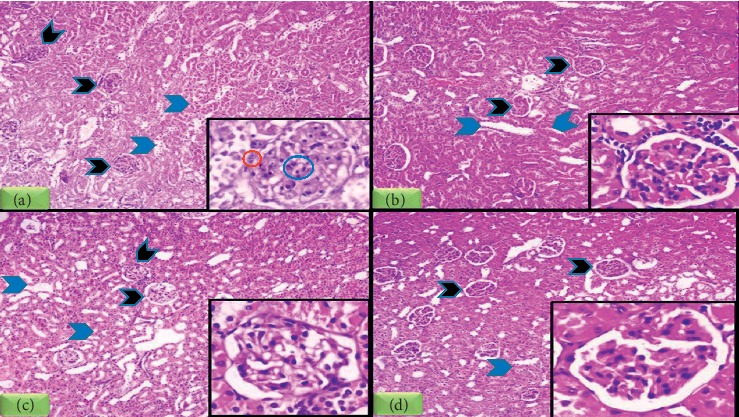
H&E stained sections of rats' kidneys in all studied group. (a) Section of DN group showed moderate hypercellular glomeruli (black arrow heads) and extensive tubular degenerative changes (blue arrow heads). Inset: high power view demonstrating mesangial proliferation (blue circle) and chronic inflammatory cellular infiltrates (red circle). (b) DN + vit D group showed mild cellular glomeruli (black arrow heads) and tubules exhibits regeneration (blue arrow heads). Inset: high power view. (c) Section of DN + Insulin group showed normocellular glomeruli (black arrow heads) and normal tubules. Inset: high power view. (d) Section of DN + insulin + vit D group showed normocellular glomeruli (black arrow heads) and normal tubules (blue arrow heads). Inset: high power view (H&E, 100x). Inset: 400x for all a, b, c, and d.

H&E stained slides of DN group showed hypercellular glomeruli and tubules, with extensive degenerative changes, and focal mesangial cellular proliferation and scattered chronic inflammatory cellular infiltrate were noted. Insulin-treated and vitamin D-treated groups showed improvement in renal pathologic changes with maximum improvement in combined insulin and vitamin D-treated group (Figures 7(a)–7(d)).

### 3.8. Immunohistochemical Expression of LC3 in Different Groups

**Figure 8:**
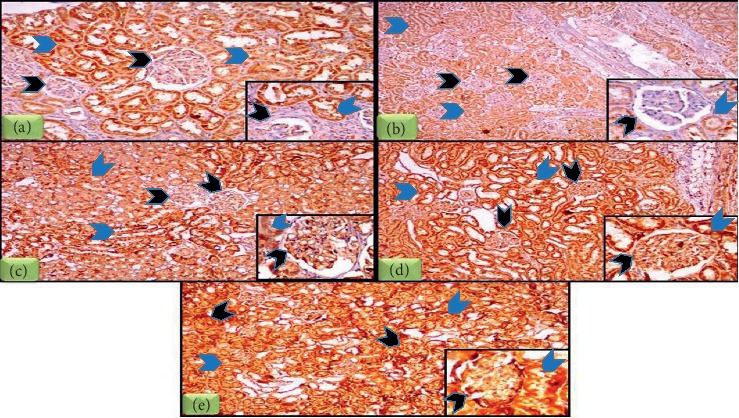
LC3 immunohistochemical staining of rats' kidneys in all studied groups. (a) Section of C group showed weak immunohistochemical expression of LC3 in glomeruli (black arrow heads) and moderate LC3 expression in tubules (blue arrow heads). Inset: high power view. (b) Section of DN group showed absence of LC3 immune reactivity in glomeruli (black arrow heads) and mild LC3 expression in tubules (blue arrow heads). Inset: high power view. (c) Section of DN + insulin showed moderate LC3 expression in glomeruli (black arrow heads) and moderate LC3 expression in tubules (blue arrow heads). Inset: high power view. (d) Section of DN + vit D group showed moderate LC3 expression in glomeruli (black arrow heads) and strong LC3 expression in tubules (blue arrow heads). Inset: high power view. (e) Section of DN + insulin + vit D group showed strong LC3 expression in glomeruli (black arrow heads) and strong LC3 expression in tubules (blue arrow heads). Inset: high power view (IHC 100x, inset 400x).

Immunohistochemical results were demonstrated in Table 1 and Figures 8(a)–8(e) which revealed that H score value of LC3 in glomeruli of DN group was significantly lower than that in control group (*P* < 0.05). H score values of LC3 in the glomeruli in insulin-treated, vitamin D-treated, and combined insulin and vitamin D-treated groups were significantly higher than those in the DN and control groups (*P* < 0.05). H score value of LC3 in glomeruli of combined insulin and vitamin D-treated group was significantly higher than those in insulin-treated and vitamin D-treated groups (*P* < 0.05). H score value of LC3 in glomeruli of insulin-treated group was significantly higher than that in vitamin D-treated group (*P* < 0.05). H score value of LC3 in the tubules of DN group was significantly lower than that in control group (*P* < 0.05). H score values of LC3 in the tubules of insulin-treated, vitamin D-treated, and combined insulin and vitamin D-treated groups were significantly higher than that in DN group (*P* < 0.05). H score value of LC3 in tubules of combined insulin and vitamin D-treated group was significantly higher than those in insulin-treated, vitamin D-treated, and control groups (*P* < 0.05). H score value of LC3 in tubules of insulin-treated group was significantly lower than those in vitamin D-treated and control groups (*P* < 0.05). H score of LC3 in tubules of vitamin D-treated group was significantly higher than that in the control group (*P* < 0.05).

## 4. Discussion

One of the serious complications of diabetes mellitus (DM) is diabetic kidney disease [21]. The result of DN group revealed significant increase in fasting blood glucose level and decrease in serum insulin. Results have been in accordance with previous reported results [22]. However, rats treated with insulin revealed significant decrease in serum glucose and increase in serum insulin when compared with DN group. These results were in agreement with previous reported results [23]. Vitamin D-treated group revealed significant decrease in serum glucose and increase in serum insulin when compared with DN group. These results were consistent with previous reported results [24]. The antidiabetic effect of vitamin D may be due to induction of insulin secretion by increasing intracellular calcium concentration and activation of *β*-cell calcium-dependent endopeptidase which facilitates the conversion of proinsulin to insulin [25]. Moreover, Wang et al. stated that vitamin D reversed insulitis and protected *β*-cells against apoptosis [26].

In our study, streptozotocin-induced diabetes in rats leads to oxidative stress evidenced by significant elevation of serum MDA level and decrease in GSH of DN group; our results are in agreement with previous reported data [27]. Hyperglycemia can lead to decline of cellular antioxidants and increased free radicals leading to an increase in lipid peroxidation and oxidative stress. Oxidative stress results in structural damage in DNA, RNA, lipids, and proteins yielding DN manifestations [28]. Insulin-treated group showed significant improvement in oxidative stress markers when compared with DN group, in agreement with previous results [29]. Treatment with vitamin D resulted in significant improvement in oxidative markers when compared with DN group. The antioxidant effect of vitamin D may be attributed to increase of the mRNA expression of some important antioxidants in the kidney [1].

Several studies revealed that the key factor for the development of DN was inflammation [30]. DN group showed significant increase in proinflammatory markers serum TNF-*α* and IL-6 levels when compared with the control group, in accordance with previous reported results [27]. TNF-*α* is the most active one in triggering the production of other cytokines such as IL-6 [31]. Albuminuria and inflammatory factors released in response to albuminuria or high glucose may all contribute to further inflammation by promoting the secretion of chemokines [32]. However, insulin-treated group showed significant decrease in proinflammatory mediators when compared with DN group. In accordance with our results, Qiu et al. reported that insulin reduced inflammation by restoring oxidative balance and decreasing the release of cytokines [33]. Our study showed that treatment with vitamin D resulted in significant decrease in TNF-*α* and IL-6 when compared to DN group. Vitamin D may promote *β*-cell survival by modulating the generation and activity of cytokines through the downregulation of nuclear factor-kappaB [34].

In our study, the combination-treated group showed significant improvement in all measured biochemical parameters when compared with DN and other treated groups. Except in oxidative stress markers, there was insignificant difference, which was attributed to the potent effects of insulin and vitamin D.

In DN group, SBP was significantly higher when compared to the control group. This result was consistent with previous reported results [35]. This could be attributed to the decrease in the levels of nitric oxide (NO) in vascular endothelium and activation of angiotensin-converting enzyme and renin angiotensin system (RAS) triggered by inflammatory effects and oxidative stress induced by DM, which increase the smooth muscle contraction and genesis of hypertension [36]. Moreover, Sourris et al. reported that AGEs induce upregulation of the RAS in the vasculature of diabetic patients that could be involved in the pathogenesis of DN [37]. SBP was significantly lower in insulin-treated group when compared to DN group. These results agree with those of Haidara et al., who mentioned that administration of insulin alone could decrease the SBP by induction of vasodilatation through its apparent ability to produce NO [38]. Vitamin D-treated group demonstrated significant decrease in SBP when compared to DN group. To date, a strong body of evidence supports vitamin D as a negative regulator of the circulating and local tissue RAS. Furthermore, the activity of vitamin D metabolites in animals is associated with reductions in blood pressure, proteinuria, and renal injury with improved *β*–cell function. Other notable hypotheses have suggested that vitamin D influences vascular endothelial function or vascular smooth muscle intracellular calcium concentrations. These results seem to suggest that vitamin D may confer cardiovascular and renal protection, especially in DN [39].

It was important, then, to identify the possible molecular mechanisms that may underlie insulin and vitamin D effects on DN. mTOR is a serine/threonine kinase and central regulator of important cellular functions. In our study, the real-time PCR results for mTOR gene demonstrated a significant upregulation of the expression of mTOR in DN group when compared to control group, which was consistent with the findings of Leventhal et al. who reported that mTOR signaling is highly activated in podocytes of diabetic kidneys [40]. Studies of mice with podocyte-specific mTOR activation induced by the conditional deletion of the upstream negative regulator TSC1 in podocytes recapitulated many features of DN, such as podocyte injury and loss, proteinuria, glomerular basement membrane thickening, mesangial expansion, and glomerulosclerosis [8]. Hyperglycemia activates mTOR primarily through the phosphatidylinositol 3-kinase/Akt signaling pathway [41]. In contrary, Ueno et al. reported that high glucose levels activate mTOR. By normalizing glucose levels, insulin therapy may deactivate mTOR [42] and this was consistent with our results in insulin-treated group. Vitamin D-treated group showed significant downregulation of mTOR expression when compared to DN group. This result was consistent with other studies [5]. This could be explained by increasing the expression of DNA-damage-inducible transcript 4 (DDIT4), which can activate tuberous sclerosis 2, and decreasing the expression of Akt, both of which result in inhibiting the expression of mTOR [43]. Blockade of the mTOR pathway reduced glomerular *α*-smooth muscle actin expression, mesangial matrix accumulation, and renal hypertrophy in STZ-induced diabetes [44]. The combined group showed significant downregulation of mTOR activity when compared with other treated groups. These findings indicate that reduction in podocyte mTOR activity protects podocytes and inhibits progressive DN, suggesting that mTOR suppression is a potential therapeutic strategy to prevent DN.

Podocytes have a limited capacity for cell division and replenishment. Autophagy is essential for cell homeostasis. Podocytes have active autophagy even under nonstress conditions, suggesting that podocytes require a high basal level of autophagy to maintain cellular homeostasis [45]. LC3 plays a key role in autophagosome biogenesis through autophagosome elongation and autophagy flux and is widely used as a sensitive index for autophagy [46]. As proved in our results, hyperglycaemia promotes oxidative stress and mitochondria are considered among the major sites of ROS in DN [40]. An impairment in the mitophagy system leads to the accelerated progression of renal pathology [47]. Immunohistochemical results showed that cell loss in renal cortex of DN group is associated with significant decrease in H score of LC3 when compared with control group, which was consistent with previous results [46]. Impaired autophagy may be involved in the pathogenesis of podocyte loss, leading to massive proteinuria with progression of DN to end-stage renal failure [48]. Hyperglycemia inhibits Jun N-terminal kinase (JNK) signaling and enhances the interaction between Beclin-1 and Beclin-2 resulting in autophagy inhibition [49]. Also, mTORC1 is an important upstream inhibitor of autophagy, and Cinà et al. reported that mTORC1 activation may be involved in autophagy inhibition in the podocytes of diabetic mice and patients [50]. Active mTORC1 inhibits autophagy by suppressing the ULK1-Atg13-FIP200 complex which is an initiator of autophagy [40]. On the other hand, insulin-treated group showed significant increase in H score of LC3 when compared with control and DN groups, which was in agreement with the findings of Han et al. who concluded that insulin treatment reversed the inhibition of autophagy present in diabetic group [51]. Vitamin D-treated group showed significant increment in H score of LC3 when compared to DN and control groups, but it was significantly lower when compared to insulin-treated group. Vitamin D could modulate autophagy via vitamin D receptor (VDR) direct regulation of gene transcription, beclin-1 and LC3. Vitamin D-induced autophagy signature was reported to be lost following VDR knockdown [13]. In addition, enhanced autophagy by vitamin D may exert renoprotective effects by promoting clearance of AGEs and preventing renal accumulation of AGEs in diabetes [52]. Expectedly, in combined group the H score of LC3 was significantly higher when compared with the other groups.

Regarding renal function tests in DN group, urinary albumin, serum urea, and serum creatinine were significantly higher when compared to control group. These results were in agreement with those of Asleh et al. who stated that podocytes were very sensitive to the toxic effect of hyperglycaemia and were primarily involved in DN with basement membrane thickening and mesangial matrix expansion with proteinuria due to alterations in the structure of podocytes [53]. Insulin-treated and vitamin D-treated groups demonstrated significant improvement in renal function tests when compared with DN group, whereas the combined group obviously showed significant improvement in renal function parameters when compared with the other treated groups, which may be attributed to the potent synergistic hypoglycemic and anti-inflammatory effects, the ability to restore the oxidative balance, and the stimulatory effect of autophagy activity of both insulin and vitamin D. In conclusion, vitamin D is an effective adjuvant therapy in the treatment of DN due to its multiple potent renoprotective effects.

## 5. Conclusion

To the best of our knowledge, this is the first study elucidating the potential effects of vitamin D combined with other antidiabetic drugs on DN. Our results obviously demonstrated that vitamin D is a potent adjuvant therapy in treatment of DN via downregulation of mTOR gene expression, stimulation of autophagy, and antioxidant, anti-inflammatory, and hypotensive effects.

## Figures and Tables

**Table 1:** H score of LC3 in glomeruli and tubules in all studied groups.

Parameters	Groups				
	C	DN	DN + insulin	DN + vit D	DN + insulin + vit D
H score of LC3 in glomeruli					
Mean ± SD	59.3 ± 3.7	0 ± 0^∗^	237.83 ± 9.9^∗#^	161.2 ± 10.8^∗#Ω^	295 ± 5.27^∗# Ω¥^
P1		<0.001	<0.001	<0.001	<0.001
P2			<0.001	<0.001	<0.001
P3				<0.001	<0.001
P4					<0.001

H score of LC3 in tubules					
Mean ± SD	205 ± 11.8	119.7 ± 6.8^∗^	181.6 ± 5.31^∗#^	274.2 ± 2.1^∗#Ω^	293.6 ± 4.5^∗# Ω¥^
P1		<0.001	<0.001	<0.001	<0.001
P2			<0.001	<0.001	<0.001
P3				<0.001	<0.001
P4					<0.05

P1: compared to C group. P2: compared to DN group. P3: compared to DN + insulin group. P4: compared to DN + vit D group (*∗*: significant when compared to C group, #: significant when compared to DN group, Ω: significant when compared to DN + insulin group, ¥: significant when compared to DN + vit D group). Data are shown as means + SD (*n* = 10). ANOVA was used to make group comparisons. Significance: *P* < 0.05.

## Data Availability

No data were used to support this study.
